# Effects of SiC Particle Size on SiC_p_/Al Composite During Vacuum Hot Pressing

**DOI:** 10.3390/ma19010084

**Published:** 2025-12-25

**Authors:** Ruijie Feng, Haibo Wu, Huan Liu, Yitian Yang, Bingbing Pei, Jianshen Han, Zehua Liu, Xishi Wu, Zhengren Huang

**Affiliations:** Ningbo Institute of Materials Technology and Engineering, Chinese Academy of Sciences, Ningbo 315201, Chinawuxishi@nimte.ac.cn (X.W.)

**Keywords:** SiC_p_/Al, microstructure, thermal property, mechanical property, particle gradation

## Abstract

High-performance SiC_p_/Al composites were fabricated via a vacuum hot-pressing powder metallurgy process. The effects of SiC particle size on the composite microstructures and their thermal and mechanical properties were systematically investigated. The vacuum hot-pressed SiC_p_/Al composites exhibited a well-bonded interface between the SiC particles and Al matrix, while not exhibiting any Al_4_C_3_ brittle phase. Particle gradation effectively enhanced the densification of SiC_p_/Al composites, resulting in dense bulks with a relative density of 99.7%. When the SiC particle size increased from 5 to 50 μm, the fracture morphologies gradually transitioned from intergranular to transgranular, while the relative density, bending strength, and thermal conductivity increased. Overall, SiC_p_/Al composites with excellent thermal conductivity (201.42 W/(m·K)) and bending strength (523 ± 29.45 MPa) were obtained. To address the scarcity of research on SiC_p_/Al composites’ thermal properties, this study establishes SiC size/gradation–property relationships, innovatively filling the gap in thermal performance regulation.

## 1. Introduction

In recent years, electronic packaging technology has become increasingly important for integrated circuits, and an ideal packaging material should exhibit a low thermal expansion coefficient, high thermal conductivity, sufficient strength and stiffness, and cost-effectiveness [1,2,3,4]. As a widely used electronic packaging material, SiC_p_/Al composites comprise either aluminum (Al) or Al alloys, with SiC particles accounting for 30 vol.% to 70 vol.% of the total content [5]. Al exhibits excellent heat dissipation properties, while SiC has a low thermal expansion coefficient. Although adjusting SiC particle content is a common way to tailor the properties of SiC_p_/Al composites, numerous studies have demonstrated that SiC particle size is another crucial factor governing the mechanical and thermal performance of the composites [6,7,8,9]. For instance, Tao et al. [8] found that larger SiC particles (14 μm) enhanced the wear resistance of Al matrix composites (wear volume loss of 5 vol.% 14 μm SiC_p_/Al was 0.05 cm^3^ at 600 N load, lower than 0.04 cm^3^ of 5 vol.% 130 nm SiC_p_/Al), while smaller particles (130 nm) contributed to higher tensile strength (210 MPa for 5 vol.% 130 nm SiC_p_/Al vs. 172 MPa for 5 vol.% 14 μm SiC_p_/Al). Liu et al. [9] further confirmed that with a fixed 30 vol.% SiC content, SiC particle size significantly affects particle dispersion uniformity, interfacial bonding quality, and defect formation: the composite with 16 μm SiC particles achieved the highest relative density (99.5%), microhardness (105 HV), and tensile strength (265 MPa), while smaller particles (6 μm) caused severe agglomeration and larger particles (23 μm) induced internal microcracks. Therefore, systematic research on the effects of SiC particle size on SiC_p_/Al composites is of great significance for optimizing their performance and expanding their application in electronic packaging.

SiC_p_/Al composites with a high SiC volume fraction (exceeding 50 vol.%) are more suitable for high-power electronic packaging due to their superior thermal management [10]. However, the poor wettability between SiC particles and molten Al hinders the achievement of high SiC volume fractions. Although increasing the temperature can slightly improve the wettability of the Al-SiC system, it also leads to the formation of a brittle and hydrolytic Al_4_C_3_ phase [11,12], which severely degrades mechanical properties. Consequently, inhibiting interfacial reactions has become critical to acquire high-performance SiC_p_/Al composites. The pressure-less infiltration method necessitates surface treatment of particles or addition of supplementary Si or Mg in the matrix alloy to suppress interfacial reactions [13,14], which are intricate and detrimental for production. Powder metallurgy, a cost-effective and highly efficient method for preparing SiC_p_/Al composites, enables the production of composites with dense structures and SiC volume fractions exceeding 60% through meticulous preparation processes and particle grading [9]. Among the various powder metallurgy techniques, hot pressing (HP) stands out as a widely employed consolidation method for fabricating high-performance metal matrix composites. Hot pressing integrates the application of temperature and uniaxial pressure simultaneously during the sintering process, which accelerates mass transfer mechanisms such as diffusion, creep, and plastic flow [15]. This synergistic effect of heat and pressure effectively eliminates residual pores, promotes intimate contact between SiC particles and Al matrix powders, and enhances interfacial bonding—addressing key limitations of conventional cold pressing followed by sintering (which often results in lower density and weaker interfaces). For SiC_p_/Al composite fabrication, hot pressing parameters (including sintering temperature, applied pressure, holding time, and heating/cooling rates) can be precisely tailored: typically, temperatures ranging from 450 to 650 °C (below the melting point of pure Al or near the solidus temperature of Al alloys) and pressures of 10 to 50 MPa are employed to avoid excessive interfacial reactions while ensuring full densification. Moreover, vacuum hot pressing (VHP), an advanced variant of hot pressing conducted in a vacuum environment, offers additional advantages: it removes adsorbed gases (e.g., oxygen, moisture) from powder surfaces, inhibits oxidation of Al powders, and reduces the formation of undesirable interfacial phases like Al_2_O_3_ and Al_4_C_3_. The vacuum atmosphere also facilitates the escape of volatile impurities, further improving the purity and uniformity of the composite microstructure. These characteristics make hot pressing, especially vacuum hot pressing, an ideal processing route for preparing SiC_p_/Al composites with high density, homogeneous particle dispersion, and enhanced mechanical-thermal synergy—critical for demanding electronic packaging applications. Nevertheless, the poor wettability between SiC and Al still results in non-uniform dispersion and weak interface binding [16]. For example, Chen et al. [10] reported that mixing 12 μm and 100 μm SiC particles could adjust the preform’s SiC volume fraction up to 68%, but fine particles (12 μm) caused pore shrinkage (to <5 μm) and required higher infiltration pressure (1.1 MPa) to ensure full impregnation. Regulating the microstructure of SiC_p_/Al composites can significantly enhance their performance. Since Al primarily exists in a molten state during sintering, microstructure regulation is primarily dependent on the bonding between SiC particles and interfaces; however, only a limited number of studies have explored how SiC particle size modulates this bonding and interface evolution, especially for high-volume fraction composites. Therefore, investigating the effects of SiC particle size on microstructure tailoring as well as the mechanical and thermal properties of SiC_p_/Al composites is also important for developing high-performance materials.

In this study, SiC_p_/Al composites with varying SiC particle sizes (5 μm, 10 μm, 20 μm, 50 μm) were fabricated via vacuum hot-pressing sintering. The primary focus was to investigate the impact of SiC particle size on the microstructure, mechanical properties, and thermal properties of SiC_p_/Al composites, clarifying the mechanism through which particle size optimizes the microstructure, such as reducing agglomeration, inhibiting Al_4_C_3_ formation, and enhancing interface bonding. To address the scarcity of research on SiC_p_/Al composites’ thermal properties, this study establishes SiC size/gradation–property relationships, innovatively filling the gap in thermal performance regulation.

## 2. Materials and Methods

### 2.1. Raw Materials

**Figure 1:**
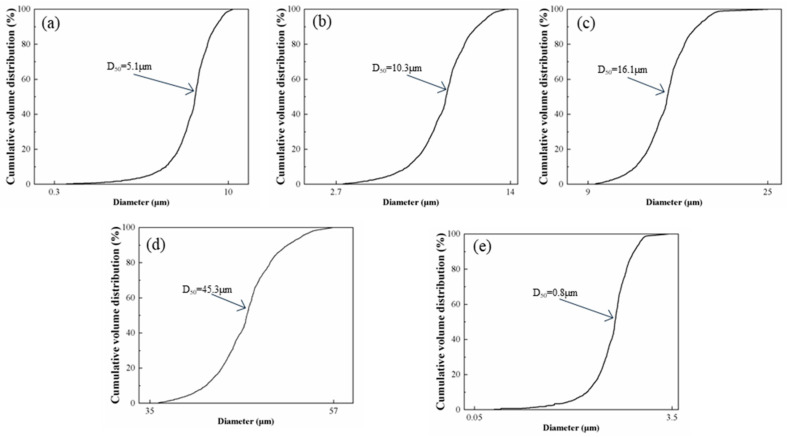
Particle size distribution curve of the raw materials: (**a**) 5 μm SiC; (**b**) 10 μm SiC; (**c**) 20 μm SiC; (**d**) 50 μm SiC; (**e**) 1 μm Al.

**Figure 2:**
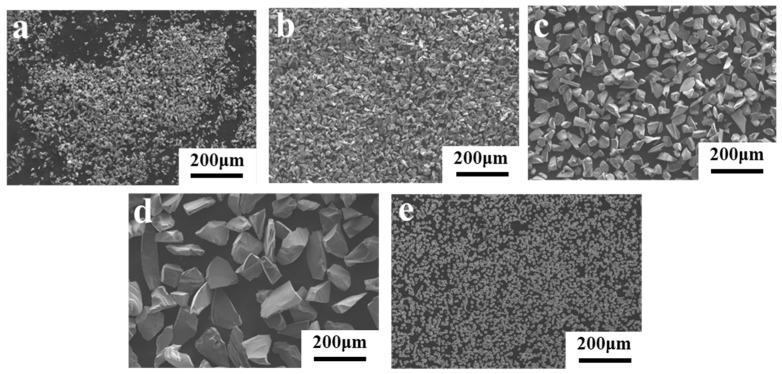
SEM images of particles used: (**a**) 5 μm SiC; (**b**) 10 μm SiC; (**c**) 20 μm SiC; (**d**) 50 μm SiC; (**e**) 1 μm Al.

High-purity Al powder (100–200 mesh, purity 4N, Hushi, Sinopharm Chemical Reagent Co., Ltd., Shanghai, China) and four types of SiC powders (purity 98.5%, Hushi, Sinopharm Chemical Reagent Co., Ltd.) with different particle sizes (purity ≥ 99%) were used as the primary raw materials, namely: (1) Al powder with a median particle size of 1 μm, (2) SiC powder with a median particle size of 50 μm, (3) SiC powder with a median particle size of 20 μm, (4) SiC powder with a median particle size of 10 μm, and (5) SiC powder with a median particle size of 5 μm. The particle size distributions and microstructures are illustrated in Figure 1 and Figure 2, respectively.

### 2.2. Vacuum Hot-Pressing Process

Samples were prepared by blending SiC powder with Al powder of varying particle sizes at a 1:1 mass ratio. Table 1 shows the properties of samples 1 to 5 (S1–S5), with 3 parallel samples prepared for each particle size to ensure experimental reliability. Subsequently, each sample powder (100 g per batch) was added to 50 mL of anhydrous ethanol (AR, Hushi, purity ≥ 99.7%, Sinopharm Chemical Reagent Co., Ltd.); ethanol was used to reduce powder agglomeration during mixing and facilitate uniform dispersion of SiC and Al particles. The mixture was subjected to ball milling for 4 h under the following parameters: rotation speed of 300 rpm, ball size of 10 mm, ball material of zirconia (to avoid contamination), and ball-to-powder mass ratio of 10:1. After ball milling, the slurry was dried at 60 °C for 8 h using a vacuum drying oven (Model DZF-6020, Jinghong, temperature range RT+10–250 °C, vacuum degree < 133 Pa), Sinopharm Chemical Reagent Co.)—vacuum conditions were adopted to accelerate ethanol volatilization and prevent oxidation of Al powder. The dried powder was passed through a 250 μm sieve to remove any large agglomerates formed during drying, ensuring consistent powder fineness for subsequent sintering and improving the uniformity of the final sintered specimen. Finally, the sieved powders underwent hot pressing and sintering in a vacuum hot-pressing furnace under a pressure of 30 MPa, temperature of 600 °C, and sintering time of 2 h to obtain the sintered specimens.

### 2.3. Material Characterization

The bulk density of the sintered sample was determined using the Archimedes method, while its relative density (RD) was calculated by determining the ratio of the measured thickness to the theoretical density via a mixture rule. Phase analysis of the composites was performed using an X-ray diffractometer (D8 Advance, Bruker, Bremen, Germany) under the following operating conditions: Cu Kα radiation (λ = 1.5406 Å) with a tube voltage of 40 kV and tube current of 40 mA; the diffraction angle (2θ) range was set from 5° to 90°, with a step size of 0.02° and a scanning speed of 5°/min. For sample preparation, the sintered specimens were ground and polished to a mirror surface to ensure a flat and clean testing surface without oxide layers. The collected diffraction data were processed using MDI Jade 6.5 software, and phase identification was conducted by matching with the standard diffraction patterns in the PDF database. The 3-point bending test of samples sized 36 mm × 4 mm × 3 mm was conducted to evaluate the flexural properties of the sintered composites using a universal testing machine (Z030TE + TEE, Zwick, Ennepetal, Germany). The test was performed with a fixed support span of 30 mm, a loading rate of 0.5 mm/min, and three parallel samples prepared for each composite type (S1–S5) to ensure data reliability. The load–displacement curves during the test were automatically recorded by the testing machine’s supporting software (MTS TestWorks 4.0). The bending strength was calculated based on the maximum load and linear elastic segment of the curves, following the calculation standards specified in the GB/T 6569-2006 [17]. Meanwhile, thermal conductivities of the composites were measured using a laser thermal conductivity meter (LFA467, NETTSCH, Selb, Germany). Prior to testing, samples were processed into a standard size of 10 mm × 10 mm × 3 mm, and their surfaces were polished to a roughness Ra ≤ 0.8 μm to minimize thermal contact resistance. The measurements were conducted at a constant temperature of 25 °C (room temperature) under a high-purity nitrogen atmosphere (purity ≥ 99.99%) to prevent sample oxidation and ensure stable heat transfer. Each sample was measured 3 times, and the average value was taken as the final result. The instrument was calibrated using GB/T 22588-2008 [18] before the test batch, and the thermal conductivity was calculated by the instrument’s built-in software (LFA Software, Version 7.0.0, NETZSCH-Gerätebau GmbH) based on the laser flash method and the Cowan model, which accounts for heat loss corrections. Furthermore, the thermal expansion coefficient (CTE) of the composites was determined using a thermal expansion meter (DIL402C, NETTSCH, Selb, Germany). Specimens were processed into a standard size of 25 mm × 5 mm × 5 mm, with both end faces ground flat and parallel to ensure accurate length measurement. The test was conducted under a high-purity nitrogen atmosphere (purity ≥ 99.99%) to avoid oxidation of the Al matrix, with a temperature range of 25 °C to 300 °C and a constant heating rate of 5 °C/min—this range and rate cover the typical service temperature of the composites and prevent thermal shock. Each sample was tested 3 times, and the average value was adopted as the final CTE result. The test process and data acquisition were controlled by NETZSCH Proteus software (Version 8.1.0), and the CTE was calculated based on the linear expansion rate of the specimen, with the software automatically correcting for the instrument’s inherent thermal expansion error. Finally, for microscopy analysis, three parallel samples were selected for each composite formulation (S1–S5) to ensure representative results. The samples were first cut into 5 mm-thick cross-sections using a low-speed diamond saw (KJ GROUP, Shenyang, Liaoning, China), then ground with 500# and 1000# sandpapers sequentially, and finally polished with 1 μm diamond paste to obtain a mirror-like surface without scratches. Cross-sectional morphology analysis of the sintered samples was conducted using a field emission scanning electron microscope (SEM, S-8230, Hitachi, Tokyo, Japan) under the following conditions: an acceleration voltage of 15 kV, a working distance of 8 mm, and a secondary electron (SE) detection method to clearly capture the surface topography and interface details. Meanwhile, metallurgical microscopy (BX51 M, Olympus, Tokyo, Japan) was employed to observe the morphology and distribution of phases in the composites, with a magnification range of 100× to 500×—100× magnification for overall phase distribution observation and 500× magnification for detailed analysis of grain boundaries.

## 3. Results and Discussion

### 3.1. Densification Behavior of SiC_p_/Al Composites

Table 1 shows that as the SiC particle size increases from 5 to 50 μm, the density of the SiC_p_/Al composites increases from 2.65 to 2.92 g·cm^−3^ and their RD increases from 90.4% to 99.7%. The low RD of the SiC_p_/Al composites with fine SiC particles can be attributed to two main factors: (1) An increase in the number of interfaces with poor wettability between SiC and Al (due to the larger specific surface area of small-sized SiC particles) results in a larger contact angle, promoting the occurrence of grain boundary porosity [19]. (2) The uneven distribution of small-sized SiC particles leads to an unbalanced composition, which further contributes to an increase in porosity. However, SiC_p_/Al composites prepared by gradating SiC particles with different sizes exhibit a relatively high density because particle gradation facilitates tight stacking, thereby reducing the pores occurring at grain boundaries due to uneven contact angles and composition distributions [20].

### 3.2. Phase Composition and Microstructure Evolution of SiC_p_/Al Composites

**Figure 3:**
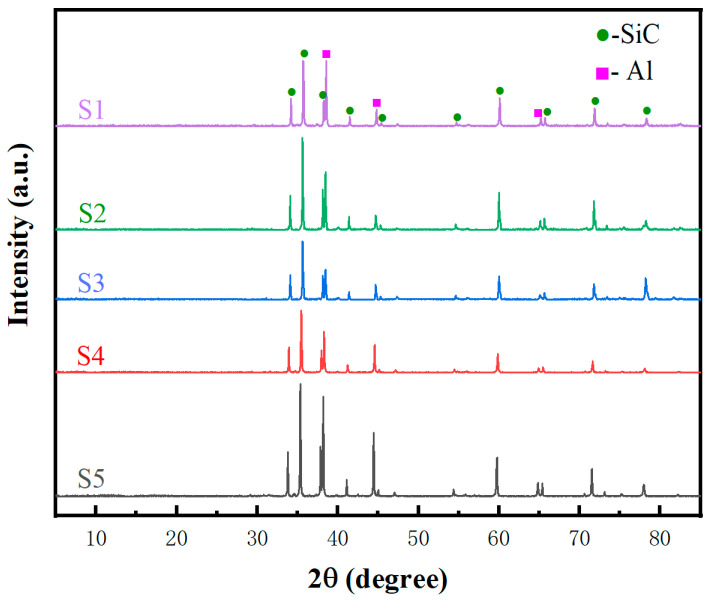
XRD patterns of SiC_p_/Al composites with different particle sizes.

Figure 3 displays the X-ray diffraction (XRD) images of SiC_p_/Al composites with different particle sizes. The XRD spectra reveal only diffraction peaks of SiC and Al, while no visible Al_4_C_3_ peak is detected; this indicates that no detectable Al_4_C_3_ was observed by XRD. Previous studies have shown that SiC_p_/Al exhibits a strong tendency to react with Al at high temperatures (Equation (1)), resulting in the formation of brittle and acicular Al_4_C_3_ [21]:

4Al + 3SiC → Al_4_C_3_ + 3Si (1)

The formation of Al_4_C_3_ has a detrimental effect on the performance of SiC_p_/Al composites. Therefore, thermodynamic calculations were performed to determine whether Al_4_C_3_ is formed via Equation (1) at the current hot-pressing sintering temperature. The Gibbs free energy was obtained using the following formula:

(2) $${\Delta_{r}G}_{m}^{\theta }={\Delta_{r}H}_{m}^{\theta }{{-T\Delta }_{r}S}_{m}^{\theta }$$

where ${\Delta_{r}G}_{m}^{\theta }$ represents the standard Gibbs free energy (KJ/mol), ${\Delta_{r}H}_{m}^{\theta }$ denotes the standard heat of reaction (KJ/mol), and ${\Delta_{r}S}_{m}^{\theta }$ signifies the standard entropy of the reaction.

The values of ${\Delta_{r}H}_{m}^{\theta }$ and ${\Delta_{r}S}_{m}^{\theta }$ involved in the thermodynamic analysis are calculated using the thermodynamic software HSC Chemistry 9.0. Equation (2) reveals that at a sintering temperature of 600 °C (i.e., 873 K), the reaction exhibits a positive Gibbs free energy change (∆G = 14.1744 KJ/mol > 0), indicating its non-spontaneity. This thermodynamic result has direct implications for the sample’s microstructure and Al_4_C_3_ formation: from the perspective of reaction tendency, ΔG^0^ > 0 means the reaction between Al and C (to form Al_4_C_3_) lacks the driving force to occur spontaneously under the experimental sintering conditions. Microstructurally, this non-spontaneity helps maintain the chemical stability of the SiC-Al interface—without Al_4_C_3_ generation, the interface bonding remains purely physical-mechanical rather than being weakened by brittle Al_4_C_3_ phases. Additionally, combined with the XRD result that no detectable Al_4_C_3_ was observed, the thermodynamic non-spontaneity further confirms that Al_4_C_3_ is not present in the sample (either below XRD detection limit or completely absent), which is crucial for ensuring the composite’s mechanical properties (e.g., bending strength) and thermal stability.

**Figure 4:**
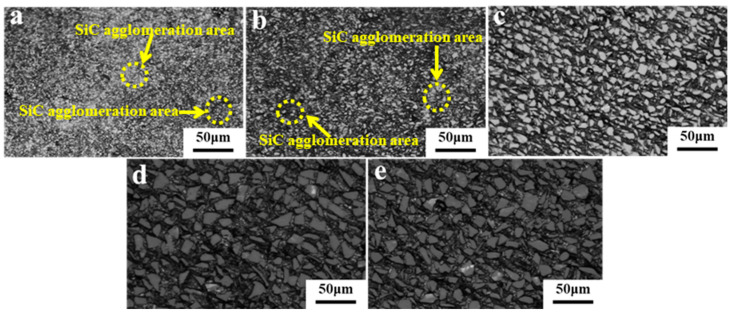
Metallographic images of SiC_p_/Al samples with different particle sizes: (**a**) S1; (**b**) S2; (**c**) S3; (**d**) S4; (**e**) S5.

Figure 4 displays the metallographic images of SiC_p_/Al composites with different SiC particle sizes. The composites with SiC particle sizes of 5 μm and 10 μm exhibit localized agglomeration, owing to the inverse relationship between particle diameter and specific surface area. A reduction in particle size leads to higher surface energy, increasing the propensity for particle aggregation. Lower densities are observed for materials with SiC particle sizes of 5 μm and 10 μm due to pore formation that occurs because of particle aggregation. In contrast, samples with particles sized 20 μm and 50 μm show uniform distribution of SiC and Al without noticeable agglomeration. Notably, in the sample comprising particles sized 50 μm, there is a greater abundance of fine SiC particles within the interstitial spaces of Al.

**Figure 5:**
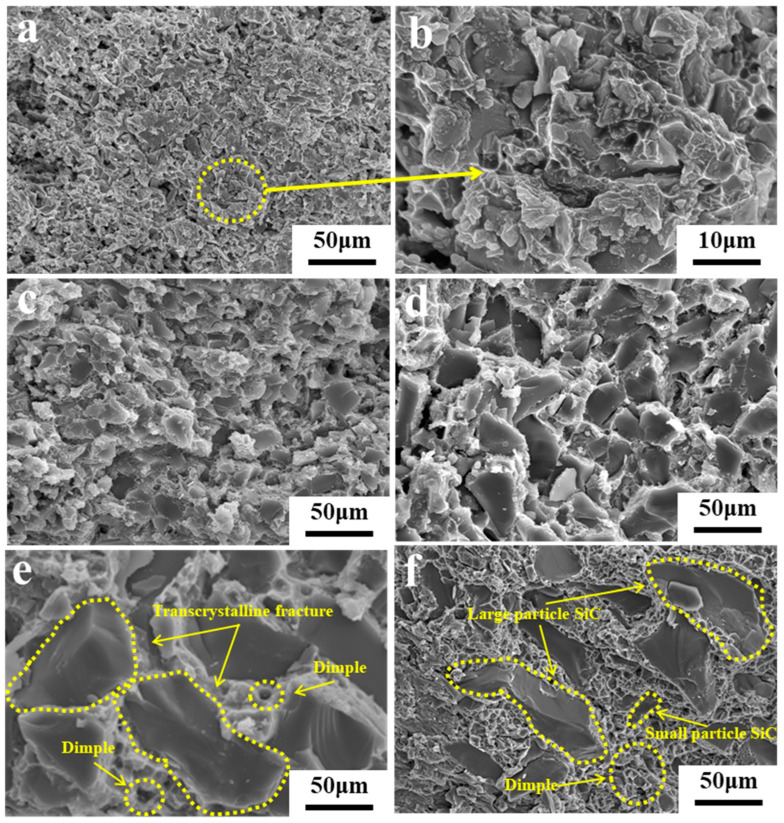
SEM images depicting the fracture morphologies of SiC_p_/Al composites: (**a**,**b**) S1; (**c**) S2; (**d**) S3; (**e**) S4; (**f**) S5.

The fracture morphologies of SiC_p_/Al samples with different SiC particle sizes are depicted in Figure 5, which indicates that larger SiC particles exhibit transgranular fracture and smaller particles undergo intergranular fracture. Additionally, dimples are observed in some regions of the Al matrix, which proves that intergranular fracture, transgranular fracture, and ductile fracture are the primary forms of fractures in SiC_p_/Al composites. Although all three types of fractures are present within the composite material, their dominance varies depending on the SiC particle size [22]. More defects and dislocations tend to accumulate within larger SiC particles, making it easier for these particles to undergo fracture. Consequently, transgranular fracture becomes the predominant mode of failure in this scenario. Conversely, the high strength and low dislocation accumulation of smaller SiC particles make them the preferred site for fracture initiation, with intergranular fracture being the primary failure mode. However, in S5 samples containing both large and small SiC particles, a tightly stacked network structure is formed, which mitigates intergranular and transgranular fractures and significantly enhances the ductile fractures characterized by dimples.

**Figure 6:**
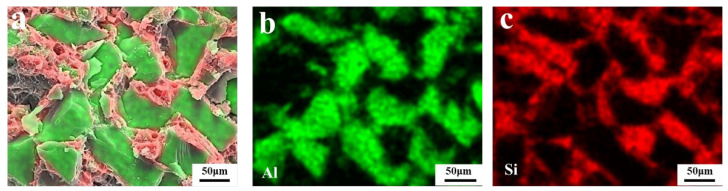
EDS images of S4 Sample: (**a**) SiC_p_/Al; (**b**) Al; (**c**) Si.

The energy-dispersive X-ray spectroscopy (EDS) images of fractures in SiC_p_/Al composites are presented in Figure 6, which demonstrate the uniform distribution of Al and SiC grains, a strong bonding between SiC and Al, and the absence of noticeable pores at grain boundaries.

### 3.3. Mechanical Properties of SiC_p_/Al Composites

**Figure 7:**
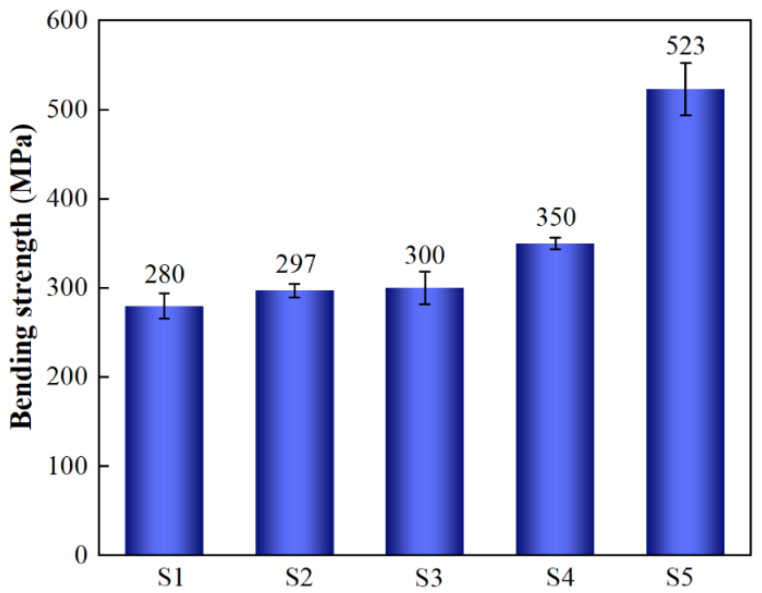
Bending strengths of different SiC_p_/Al samples.

The bending strength of SiC_p_/Al composites increased with increasing SiC particle size (Figure 7). Notably, the highest bending strength (523 MPa) was achieved in the sample with particle grading (S5). To analyze this phenomenon, it is imperative to examine the three fracture forms observed during the fracture process of composites [22]: (1) intergranular fracture of tiny particles, (2) transgranular fracture of large particles, and (3) ductile fracture characterized by dimples between the Al matrix. These observations and SEM images of the fractures reveal that as the size of reinforcing particles increases, the fracture mode gradually transitions from intergranular to transgranular. This transition confirms that smaller particles can endure higher loads due to their limited defects and thermal mismatch strengthening, which cause cracks to deflect along grain boundaries and consequently increase bending strength. Conversely, larger particles exhibit transgranular fractures, which have an inverse effect on bending strength. Thus, under normal circumstances, bending strength decreases with increasing particle size. The above-mentioned experimental results can be attributed to several factors: (1) low densities of S1 and S2 samples, (2) residual stress caused by thermal mismatch, and (3) the impact of Al and SiC distribution uniformity on material properties. First, a low RD inevitably leads to a decline in material performance and is primarily responsible for the reduced bending strength observed in S1 and S2 samples. Second, with an increase in the SiC particle size, the residual stress caused by thermal mismatch diminishes [23], thereby enabling SiC particles to bear more loads and thus enhancing bending strength. Meanwhile, a better distribution uniformity of larger SiC and Al particles facilitates the development of a network structure formed by Al that evenly distributes loads throughout the material, while preventing preferential crack propagation at some locations [24]. Therefore, when considering the influence of particle size on bending strength, it is crucial to consider the above-mentioned three factors collectively. The exceptional load-bearing ability of small SiC particles surpasses the impact of residual stress caused by thermal mismatch and the crack propagation occurring due to uneven distribution; thus, increasing the bending strength causes the SiC particle size to decrease. Conversely, larger SiC particles exhibit a reduced load-bearing ability due to defects and dislocation accumulation, thus leading to transgranular fracture and a decrease in the bending strength. However, with an increase in the SiC particle size, changes in the bearing capacity of the particles become less evident at this stage. Meanwhile, the rapid decrease in residual stress caused by thermal mismatch and the enhanced distribution uniformity result in uniform load bearing, thereby preventing preferential crack formation and thus increasing the bending strength of the material. Therefore, S4 demonstrates a higher bending strength than S3, which can also be deduced from the fracture topography shown in Figure 4; that is, an increase in the number of dimples on the Al matrix indicates enhanced toughness, which is closely associated with an improved bending strength. Particle grading in SiC_p_/Al composites enables efficient load transfer under tight stacking conditions, and SiC particles with smaller diameters can carry higher loads. Additionally, SiC particles with larger diameters mitigate residual stress caused by thermal mismatch and enhance particle uniformity within the material matrix. Consequently, a ~25% improvement in bending strength is achieved for particle-graded SiC_p_/Al composites, reaching a value as high as 523 ± 29.45 MPa.

### 3.4. Thermal Properties of SiC_p_/Al Composites

**Figure 8:**
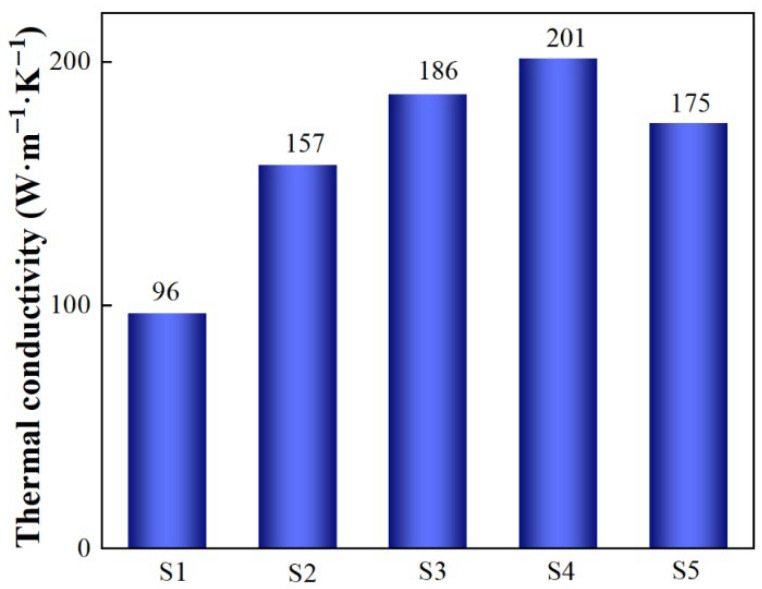
Thermal conductivity of different SiC_p_/Al samples.

The thermal conductivities of SiC_p_/Al composites prepared with different SiC particle sizes are shown in Figure 8. As the SiC particle size increases, the thermal conductivity of the composite also increases; S4 shows the highest thermal conductivity at 201.42 W/(m·K). Electronic thermal conductivity is the primary heat transfer mechanism in Al, while phonon thermal conductivity is responsible for heat transfer in SiC. Therefore, the thermal conductivity of SiC_p_/Al composites is dependent on both Al and SiC [25]. In small SiC particles, there are more interfaces between SiC and Al, which lead to a higher interface thermal resistance and a significantly lower thermal conductivity of SiC_p_/Al composites. However, in large SiC particles, the influence of interface thermal resistance on thermal conductivity diminishes significantly. Meanwhile, because a combination of particles sized 5 μm and 50 μm was used during the particle grading process, the resulting thermal conductivity falls between that obtained for materials prepared with only particles sized either 5 μm or 50 μm. Since some smaller and larger particles connect to form a continuous phase within this structure, interfacial resistance reduces, leading to a lower overall interface thermal resistance compared to that observed in composites prepared solely with particles sized 50 μm; this results in a slightly reduced thermal conductivity of 175.43 W/(m·K).

**Figure 9:**
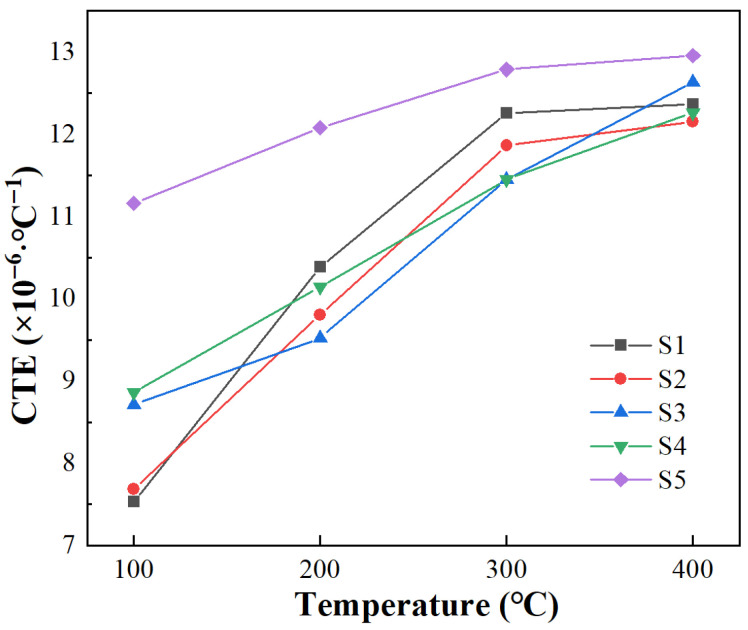
CTE of different SiC_p_/Al composites.

The average coefficient of thermal expansion (CTE) of SiC_p_/Al composites prepared using SiC particles of different sizes is presented in Figure 9, illustrating the variation observed in CTE from room temperature to higher temperatures. CTE increases significantly with an increase in particle size. Since the CTE of Al (23.21 × 10^−6^/K) is higher than that of SiC (4.7 × 10^−6^/K), it undergoes expansion in composite materials. Generally, the expansion of metals is primarily attributed to an increase in atomic spacing. However, due to the presence of SiC particles, the expansion of Al is restricted by SiC particles passing through the interface between SiC and Al, resulting in thermal mismatch stress [23]. When the thermal mismatch stress exceeds the yield strength of Al, plastic deformation of Al is required to release stress. However, since SiC particles do not undergo deformation easily, the plastic deformation of Al is restricted as well; this leads to significant accumulation of dislocations at SiC particles until both the thermal mismatch stress and accumulated dislocations exceed the yield strength of SiC and force its deformation. The size of SiC particles directly influences the required yield strength for deformation. Smaller SiC particles result in higher yield strength, which has dual effects: first, it better restricts Al deformation and reduces the thermal expansion coefficient; second, it leads to more significant thermal mismatch stress. This stress cannot be fully released during cooling due to the increased difficulty in plastic deformation because of temperature reduction, thus resulting in residual stress within the material. This result shows that smaller SiC particles lead to higher residual stress levels. CTE also increases with elevated temperatures, which cause the destruction of the SiC-Al interface and weaken the restraining effect of SiC on Al. Compared to SiC_p_/Al composites prepared using a single SiC particle size, the CTE of composites prepared with particle gradation is relatively higher. This can be attributed to the formation of a continuous SiC phase in S5, which leads to the SiC particle becoming large in the thermal expansion process, thereby facilitating the thermal expansion of Al. Additionally, the reduced spacing between SiC particles significantly impedes dislocation slip, leading to a higher density of retained dislocations within the composite material and a correspondingly larger internal stress. Consequently, atomic energy within the material increases and its nuclear activity at the same temperature increases, thereby contributing to its more significant CTE [25]. The thermal expansion coefficient of SiC_p_/Al composites prepared via SiC particles sized 5 μm reaches 7.5349^−6^/K at 25–100 °C.

## 4. Conclusions

In summary, high-performance SiC_p_/Al composites with different SiC particle sizes were vacuum hot-pressed, and the effects of SiC particle size on the microstructure and properties of these composites were investigated. The following conclusions were drawn from this study:

(1) With an increase in the SiC particle size, the fracture morphologies of the composites gradually transition from intergranular to transgranular. Owing to the presence of SiC particles with varying sizes, both transgranular and intergranular fracture characteristics are observed during particle grading. EDS images demonstrate that SiC and Al are uniformly distributed in the composite, with no Al_4_C_3_ formed at the interface and no noticeable porosity observed at the grain boundaries.
(2) The bending strength of the composites increases with increasing SiC particle size, which can be attributed to the combined effects of RD and residual thermal stress. Notably, the particle-graded sample (S5=) exhibits the highest flexural strength because particle grading in SiC_p_/Al composites enables efficient load transfer under tight stacking conditions, with smaller SiC particles taking on higher loads. Additionally, larger SiC particles mitigate residual stress from thermal mismatch and enhance particle uniformity within the material matrix.
(3) Thermal conductivity increases with a rise in the SiC particle size because larger SiC particles form fewer interfaces than smaller SiC particles, and the existence of an interface significantly reduces thermal conductivity. CTE increases with a rise in SiC particle size because smaller SiC particles have a more restrictive effect on the thermal expansion of Al through the interface.

## Figures and Tables

**Table 1:** Properties of SiC_p_/Al composites.

Sample	Particle Sizes of SiC (μm)	Bulk Density (g·cm^−3^)	Theoretical Density (g·cm^−3^)	Relative Density (%)
S1	5	2.65	2.93	90.4
S2	10	2.77	2.93	94.5
S3	20	2.81	2.93	95.9
S4	50	2.92	2.93	99.7
S5	5 + 50	2.92	2.93	99.7

## Data Availability

The original contributions presented in this study are included in the article. Further inquiries can be directed to the corresponding authors.
